# Enzymatic properties of UDP-glycosyltransferase 89B1 from radish and modulation of enzyme catalytic activity via loop region mutation

**DOI:** 10.1371/journal.pone.0299755

**Published:** 2024-02-28

**Authors:** Hiroyuki Ohashi, Daisuke Koma, Hayato Yamanaka, Takashi Ohmoto

**Affiliations:** Osaka Research Institute of Industrial Science and Technology, Osaka-City, Osaka, Japan; University of Mississippi, UNITED STATES

## Abstract

Glycosyltransferases (GTs), crucial enzymes in plants, alter natural substances through glycosylation, a process with extensive applications in pharmaceuticals, food, and cosmetics. This study narrows its focus to GT family 1, specifically UDP-glycosyltransferases (UGTs), which are known for glycosylating small phenolic compounds, especially hydroxybenzoates. We delve into the workings of *Raphanus sativus* glucosyltransferase (Rs89B1), a homolog of *Arabidopsis thaliana* UGT89B1, and its mutant to explore their glycosyltransferase activities toward hydroxybenzoates. Our findings reveal that Rs89B1 glycosylates primarily the *para*-position of mono-, di-, trihydroxy benzoic acids, and its substrate affinity is swayed by the presence and position of the hydroxyl group on the benzene ring of hydroxybenzoate. Moreover, mutations in the loop region of Rs89B1 impact both substrate affinity and catalytic activity. The study demonstrates that insertional/deletional mutations in non-conserved regions, which are distant from the UGT’s recognition site, can have an effect on the UGT’s substrate recognition site, which in turn affects acceptor substrate selectivity and glycosyltransferase activity. This research uncovers new insights suggesting that mutations in the loop region could potentially fine-tune enzyme properties and enhance its catalytic activity. These findings not only have significant implications for enzyme engineering in biotechnological applications but also contribute to a more profound understanding of this field.

## Introduction

Glycosyltransferases (GTs), enzymes integral to the biosynthesis of biologically active substances in plants, catalyze the transfer of sugar molecules from nucleoside diphosphate donors to a broad spectrum of acceptor molecules, thereby forming glycosidic bonds. This process, termed glycosylation, alters the physical properties and functions of the organic compounds, or aglycon, which are the substrates for sugar addition. Various methodologies for this process have been explored [1]. A key advantage of GTs is their high selectivity in forming glycosidic bonds with minimal byproducts, simplifying the downstream purification of high-purity products. As a result, there is a growing interest in discovering highly active enzymes and applying protein engineering techniques to modify their functions [2]. Furthermore, GTs are coveted for their potential industrial applications. The glycosides produced through GT-catalyzed reactions exhibit altered physical properties and may display different biological activities compared to aglycon [3], making them promising candidates for diverse applications in industries such as pharmaceuticals, food, and cosmetics. Therefore, comprehending the mechanisms and functions of GTs is crucial for leveraging their potential for industrial use, and research continues to concentrate on exploring and optimizing these enzymes utilization in different applications [4].

GTs are categorized into 116 distinct families in the carbohydrate-active enzymes database (CAZy), based on factors such as sequence similarity, substrate recognition, and phylogenic analysis [5]. GT family 1, which contains the largest number of GTs found in plants, is of particular interest. Most members of this family possess a C-terminus consensus sequence known as the plant secondary product glycosyltransferase (PSPG) box. The PSPG box is presumed to be the region recognizing a uridine 5’-diphosphate (UDP)-sugar [6, 7]. Typically, GTs in this Family are UDP-glycosyltransferases (UGTs) that utilize low-molecular weight organic compounds as acceptor substrates and recognize uridine 5’-diphosphate (UDP) sugars as donor substrate [4, 6, 7]. Amino acid residues in close proximity to the PSPG box in the steric structure are postulated to be responsible for the glycosylation to organic compounds recognized by the enzyme pocket, but the details are still unknown [6, 7].

The glycosylation of benzoates, a class of natural products with significant industrial and strategic value, has been thoroughly studied in plants. In *Arabidopsis thaliana*, a model plant species, 14 UDP-glucosyltransferases from GT family 1 have been associated with the glucosylation of benzoates [8]. These reactions lead to the formation of benzoate conjugates, where sugars or carboxyl groups are attached to specific hydroxyl groups on the benzene ring. This modification can drastically change the properties and functions of benzoates, making them crucial intermediates in various metabolic pathways [6].

The industrial significance of benzoate glucosylation lies in the potential applications of benzoic acid glycosides. The process of glycosylation can enhance the volatility, stability, and bioavailability of benzoates [9], offering considerable industrial benefits. Moreover, glycosylated benzoates exhibit altered bioactivity, such as antibacterial properties, compared to their unglycosylated counterparts. This opens up new avenues for the discovery of novel bioactive compounds for a wide range of industrial applications [10].

For industrial applications of glycosyltransferases, various mutations have been introduced into the enzyme to modify its enzymatic properties. Specifically, efforts have been made to alter substrate recognition and glycosylation sites. Techniques to change enzyme activity by substituting amino acid residues involved in substrate recognition sites or in the formation of glycosidic bonds in enzymes have been employed [11–14]. Recent, attempts have been made to enhance substrate reaction specificity by focusing on the loop region connecting the units of the enzyme and optimizing the amino acid sequence of this region [15]. However, the correlation between the introduction of mutations in the loop region and changes in enzyme activity is still unclear, and further research is needed for efficient protein engineering using loop region modification as a potential tool for modifying enzyme properties.

**Fig 1:**
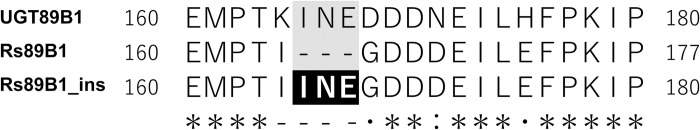
Annotated alignment of UGT89B1, Rs89B1, and Rs89B1_ins. Amino acid sequence alignment was performed using the Clustal Omega multiple sequence alignment program. The complete alignment results are presented in S1 Fig. At the bottom of the sequence, * denotes a conserved sequence (identical),: denotes a conservative mutation, denotes a semiconservative mutation, and–denotes a gap.

In this study, we focused on the non-conserved amino acid sequence region between the UDP-glycosyltransferase of *A*. *thaliana* (UGT89B1) and the putative UDP-glycosyltransferase of *Raphanus sativus* (Rs89B1), a homolog of UGT89B1, to explored the effect on the properties of the enzyme. We hypothesized that the insertion a few amino acid residues in a non-conservative region might affect enzyme activity and substrate affinity. To test this hypothesis, we constructed Rs89B1_ins, a mutant Rs89B1 with site-directed insertional mutagenesis in the loop region where the amino acid sequences of UGT89B1 and Rs89B1 differ (Fig 1). We then compared the properties of Rs89B1 and Rs89B1_ins through biochemical analyses, including enzymatic characterization and enzyme kinetic measurements. The results revealed differences in substrate affinity and enzyme catalytic activity between Rs89B1 and Rs89B1_ins, indicating that the insertion of a few amino acid residues can lead to changes in enzyme properties.

In conclusion, this study illuminates the differences in substrate affinity and enzyme catalytic activity between Rs89B1 and Rs89B1_ins, enzymes corresponding to UGT89B1. It provides valuable insights into the molecular basis of substrate recognition and the enzyme activity of GT family 1 enzymes. The findings enhance our understanding of plant GTs and their role in hydroxybenzoate glycosylation, with implications for enzyme engineering and biotechnological applications. Further research could lead to the development of novel enzymes with tailored substrate specificity and enhanced catalytic efficiency for industrial applications.

## Material and methods

### Materials

All chemicals and reagents used in this study were of special or biochemical grade and sourced from the specified suppliers. The following compounds were obtained from Tokyo Kasei Kogyo Co. (Tokyo, Japan): 2-hydroxy benzoic acid (2-HBA), 2,3-dihydroxy benzoic acid (2,3-DHBA), 2,5-DHBA, 2,6-DHBA, 3,5-DHBA, 2,3,4-trihydroxy benzoic acid (2,3,4-THBA), and 2,4,6-THBA. 2,4-DHBA and 3,4-DHBA were procured from Wako Pure Chemical Industries, Ltd. (Osaka, Japan). 3-HBA was sourced from Nacalai Tesque Inc. (Kyoto, Japan). 4-HBA was obtained from Kishida Kagaku Co., Ltd. (Osaka, Japan). UDP-d-glucose was supplied by Yamasa Co., (Chiba, Japan).

### Homology search and protein sequence alignment of UGT89B1

Protein-protein Basic local alignment search tool (BLAST) was utilized to search the nonredundant protein sequence (nr) database for a putative gene encoding a glucosyltransferase protein [16]. The search was performed using UGT89B1 (GenBank Protein ID, NP_177529.2) as a reference sequence, except for *Arabidopsis* (taxid:3701). Amino acid sequences belonging to the five taxonomies were picked out in descending order of total score. Multiple amino acid sequence alignment of the picked up and Rs89B1_ins was performed using the Clustal Omega multiple sequence alignment program [17].

### Cloning of putative *R*. *sativus* UGT89B1 (Rs89B1)

The full-length CDS of the putative *Rs89B1* gene (GenBank Gene ID, 108814497) was amplified using the primer set (Rs89B1_F; 5’–TATAGTCGCTTTGTTAAATCATATGACGGTCAACGAGGAAAACAC–3’, Rs89B1_R; 5’–GTGATGGTCGACGGCGCTATTCTTCTTCTGCCCTAAGCTAACGATA–3’, underlined parts indicate overlap regions with pREP1-His vector) from *R*. *sativus* cDNA. The PCR amplification was performed using PrimeSTAR Max DNA Polymerase (TaKaRa, Shiga, Japan) under conditions according to the instruction manual.

Prior to the preparation of the linear fragment of vector, pREP1-His vector was prepared by inserting the 6 × His tag sequence into the multiple cloning sites of pREP1 vector [18]. pREP-His vector was constructed using PrimeSTAR Max mutagenesis basal kit (TaKaRa, Shiga, Japan). PCR amplification was performed under the conditions of the instruction manual using the primer set (REP-His1; 5’–**GATGGTGATG**GTCGACGGCGCTATTCATATGATTTAACAAAGCGAC–3’, REP-His2; 5’–TCGAC**CATCACCATC****ACCATCAC**TGACTCTAGAGGATCCCCG–3’, bold parts indicates the inserted *6 × his* and underlined parts indicates overlapped regions) and then transformed into Escherichia coli DH5α strain. A linear fragment of the pREP1-His vector was amplified with PrimeSTAR Max DNA polymerase using the primer set (REP_F; 5’–AATAGCGCCGTCGACCATC–3’, REP_R; 5’–CATATGATTTAACAAAGCGACTATAAGTC–3’) and under the reaction conditions in the instruction manual.

The *Rs89B1* PCR fragment was then assembled with the linear fragment of pREP1-His using the NEBuilder HiFi DNA Assembly technique (New England Biolabs, Ipswich, MS, USA), resulting in the creation of pREP1-Rs89B1_His. This expression vector was then introduced into the *Schizosaccharomyces pombe* TN4 strain (*h*^*−*^ *leu1-32*) using the lithium acetate method [19].

### Site-directed insertional mutation in Rs89B1

Mutational introduction into the loop region of Rs89B1 was performed by site-directed insertional mutation using PrimeSTAR Max mutagenesis basal kit. pREP1-Rs89B1_ins was constructed by PCR-amplification using PrimeSTAR Max PCR mix, pREP1-89B1_His as a template, and site-specific primers (InsR: 5’–TCCTTCGTTGATGATGGTGGGCATCTCGATCC–3’, InsL; 5’–ATCATCAACGAAGGAGACGACGATGAGATACTCGAG–3’, underlined parts indicate overlap regions). The PCR amplification condition was in accordance with the product instruction. This expression vector was then also introduced into the *S*. *pombe* TN4 strain using the lithium acetate method [19].

### Expression and purification of His-tagged proteins

*S*. *pombe* transformants were grown for 72 h using EMM-leu medium [20]. The cultivated cells were collected at 3,000 × *g*, and the cell pellets were rinsed with ice-cold distilled water. The cells were then lysed using acid-washed 0.5 mm diameter glass beads in 45 cycles of 30 sec vortexing and 30 sec cooling on ice with an extraction buffer (50 mM Tris-HCl pH 7.5, 0.3 M NaCl, 1.0 mM phenylmethylsulfonyl fluoride (PMSF)). Glass beads, unbroken cell, and cell debris were removed from cell lysates by centrifugation at 10,000 × *g* at 4°C. The supernatant was then loaded onto a HisTrap HP 1 mL column (Cytiva, MA, USA). The washing step was performed with 10 column volumes of washing buffer (50 mM Tris-HCl pH 7.5, 0.3 M NaCl, 1 mM PMSF). Affinity-bound proteins were eluted with three-column volumes of washing buffer containing 300 mM imidazole. The elution fractions were desalted and substituted for storage buffer (50 mM Tris-HCl pH 7.5, 1 mM MgCl_2_, 50% (v/v) glycerol) using a PD-10 column (GE Healthcare, NJ, USA) and stored at −20°C.

### Glucosyltransferase activity assay

The Rs89B1 assay was adapted from a previously described method [8, 21]. A basic glucosyltransferase assay mix (400 μL) was prepared, consisting of approximately 0.1 nkat of recombinant protein, 50 mM sodium phosphate buffer at pH 7.0, 0.5 mM 2-mercaptoethanol, 5.0 mM UDP-glucose, and 1.0 mM of hydroxybenzoate. The specific activities were determined by obtaining time courses using 5.0 mM 4-HBA and UDP-glucose at 30°C and pH 7.0, using 0.1 mg/mL of purified Rs89B1 or Rs89B1_ins. Reaction solutions that did not include the enzyme were preincubated at 30°C for 5 min, after which the enzyme was added to initiate the enzymatic reaction. The reaction proceeded at 30°C for 10 min and was terminated by adding 1/20 of the volume of 1% HCl. The samples were stored at −20°C before HPLC analysis. The quantities of glucosides generated during the enzymatic reaction were determined using a calibration curve for aglycones.

Enzyme kinetic parameters for benzoates were assessed over a range of 0.025 to 5.0 mM in the presence of a glycosyltransferase assay mix (100 μL) containing 0.02 nkat of Rs89B1 or Rs89B1_ins. Kinetic parameters for UDP-glucose were also determined across the range of 0.02 to 10.0 mM, using 1.0 mM 2,3,4-THBA as an acceptor substrate. Reactions were carried out at 30°C for 5 min. The initial reaction rate was measured in three replicates for each substrate concentration, enzyme kinetic parameters were then calculated using curve fitting.

### HPLC analysis

Enzyme-reacted samples were diluted fivefold with a solution of 30% methanol and 0.1% formic acid. Subsequently, 5 μL of the diluted samples were used for analysis. The HPLC analysis was conducted using a Shimadzu Nexera XR system. For phenolic compounds, the HPLC conditions were as follows: The column used was COSMOSIL PBr (4.6 mm inner diameter × 150 mm, 5 μm particle size, Nacalai Tesque); the eluent was isocratic with a mixture of 0.1% (v/v) formic acid and methanol at a ratio of 70:30 (v/v); the flow rate was set to 1.0 mL/min; the column temperature was maintained at 40°C; and detection was performed at wavelengths of 250 nm, 310 nm, and 300 nm with a 4 nm bandwidth.

## Results

### Screening for *R*. *sativus* genes with homology to UGT89B1

Sequences were extracted from the top five taxonomic groups based on their total score in the BLAST search results and multiple alignments were then performed using Clustal Omega. The largest proportion of the amino acid sequences compared showed high homology to UGT89B1, but low homology was observed in the region of amino acid residues 164–168 of UGT89B1. In particular, sequences derived from *Capsella rubella* (NCBI reference sequence, XP_023642898.1), *Raphanus sativu* (NCBI reference sequence, XP_018442586.2), and *Brassica carinata* (NCBI reference sequence, KAG2322519.1) were found to have three missing amino acid residues (S1 Fig and Fig 1). The deletion residues were located far from the conserved region, which was presumed to be the PSPG box. An investigation was carried out into the function of the mutation using the putative UDP-glucosyltransferase from the *R*. *sativus* sequence among the aligned sequences. For further analysis, the mutant enzyme Rs89B1_ins was constructed with reference to UGT89B1. This enzyme is an enzyme with three amino acid residues inserted between the 164th and 165th amino acid residues of Rs89B1 (Fig 1). The structures of Rs89B1 and Rs89B1_ins were inferred using AlphaFold2 [22, 23] with predicted structure of UGT89B1 as a template structure, the gap/mutated residues were found to correspond to a loop region that connects protein domains (S2 Fig).

### Substrate selectivity of Rs89B1 and Rs89B1_ins

**Fig 2:**
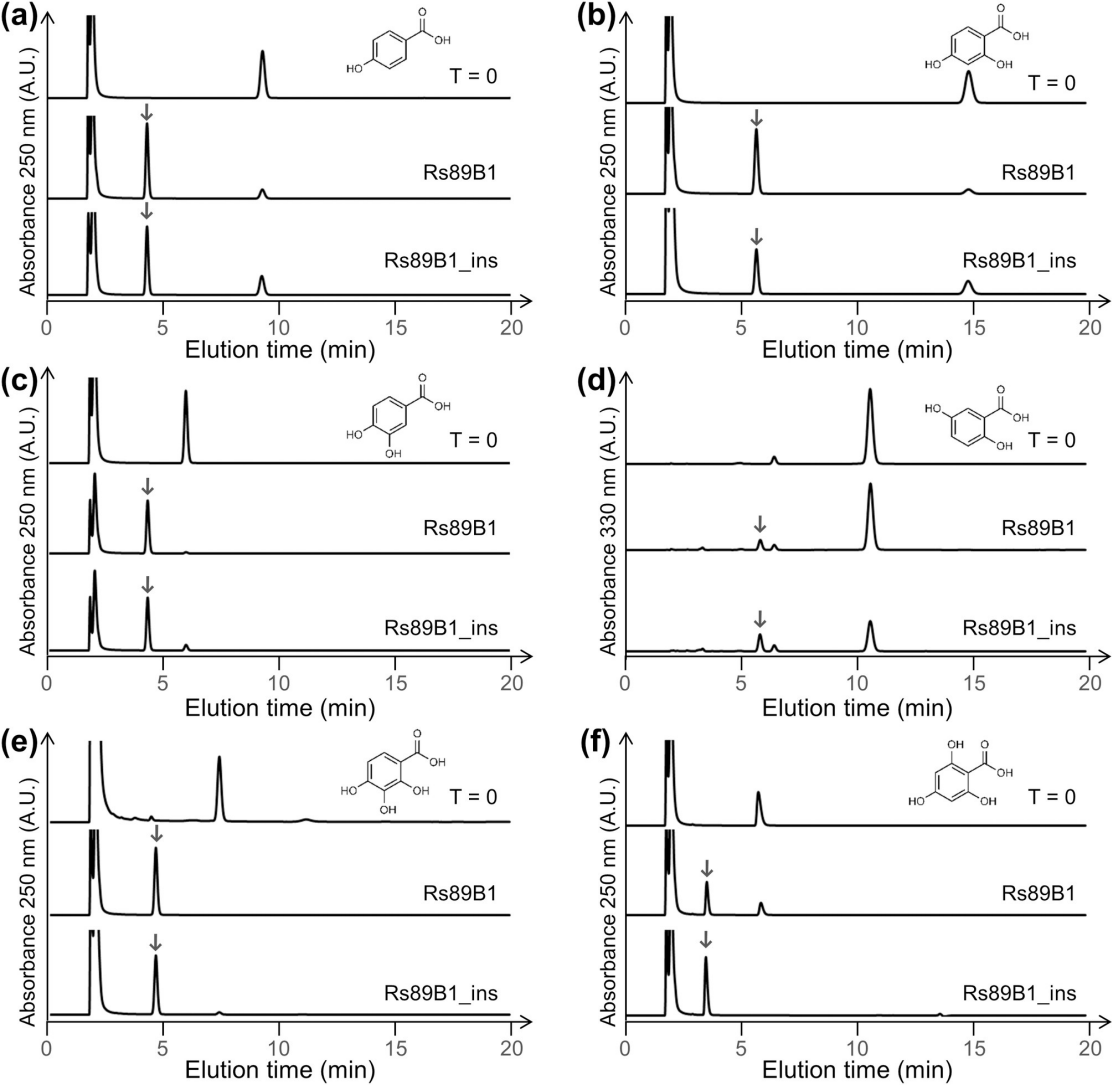
Activity of Rs89B1 and Rs89B1_ins on hydroxyl benzoates. Results of HPLC analysis of the enzyme reaction products. Substrates where the enzyme was active, including 4-HBA (a), 2,4-DHBA (b), 3,4-DHBA (c), 2,5-DHBA (d), 2,3,4-THBA (e), and 2,4,6-THBA (f), are displayed. Substrates for which no enzymatic activity was detected are presented in S5 Fig. The downward arrow (↓) indicates the enzyme reaction products.

Rs89B1 and Rs89B1_ins were expressed as C-terminal His-tag fusion proteins in *S*. *pombe*, serving as the expression hosts, and the purified enzymes were employed for analysis (S3 Fig). An enzyme unit was defined as the amount of enzyme that produces 1 mol of 4-hydroxybenzoate-d-glucoside from 5.0 mM 4-HBA in 1 second at 30°C, pH 7.0. Rs89B1 and Rs89B1_ins exhibited activities of 17.1 nkat/mg-protein and 14.2 nkat/mg-protein, respectively (S4 Fig). We conducted an analysis of the activity against structurally related hydroxybenzoates (2-HBA, 3-HBA, 4-HBA, 2,3-DHBA, 2,4-DHBA, 2,5-DHBA, 2,6-DHBA, 3,4-DHBA, 3,5-DHBA, 2,3,4-THBA, and 2,4,6-THBA) (Fig 2 and S5 Fig). In structures with a hydroxyl group at the *para*-position of benzoic and 2,5-DHBA, novel peaks were detected through enzymatic reactions. The *m/z* values of the novel peaks shifted from the *m/z* of the substrate by *m/z* 162.052, indicating hexose transfer (S1 Table). Interestingly, glycosyltransferase activity towards 2,3,4-THBA and 2,4,6-THBA was newly detected, indicating that analogous enzymes of Rs89B1, such as UGT89B1, are also capable of glycosylating THBAs.

### Effects of temperature, pH and divalent cations of Rs89B1 and Rs89B1_ins

**Fig 3:**
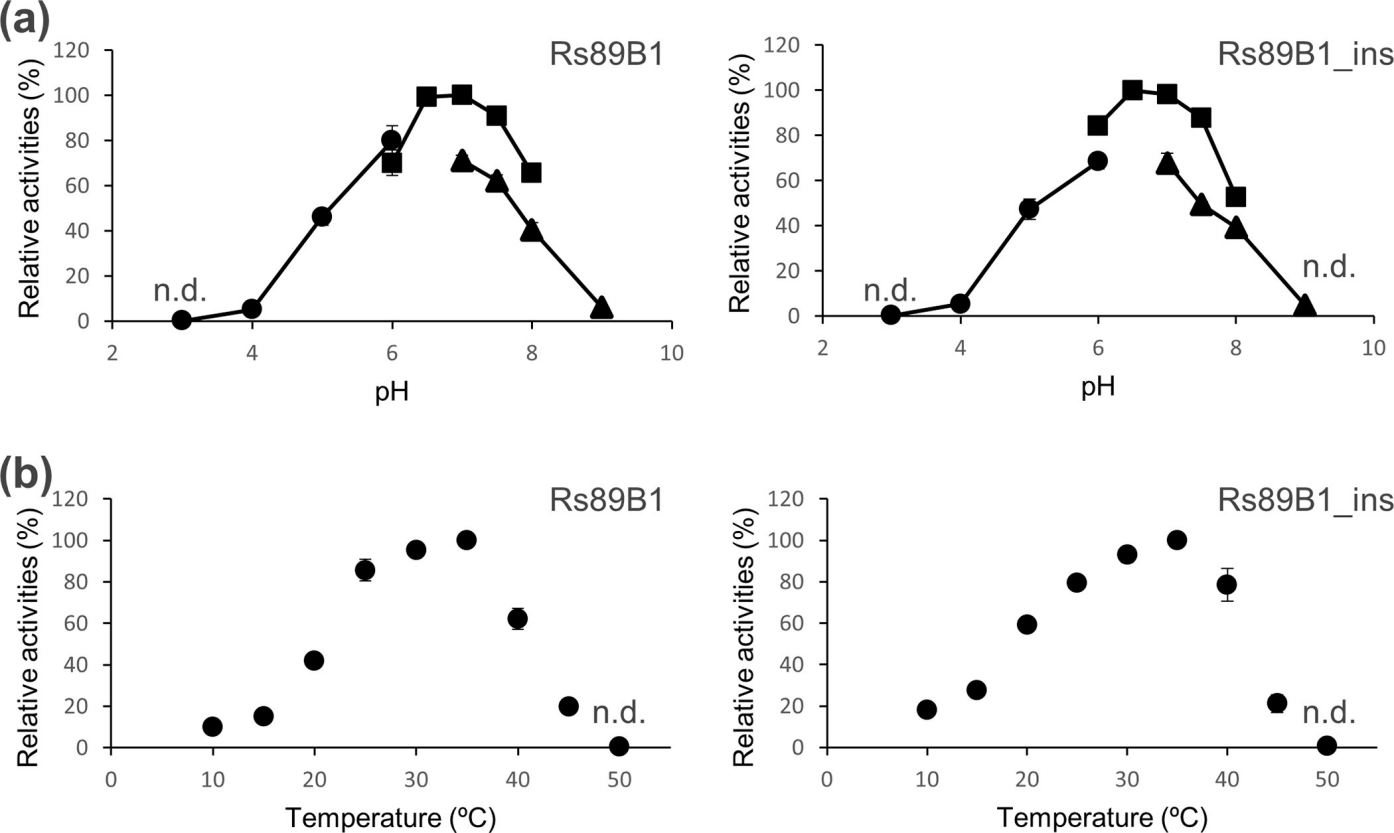
Effects of pH and temperature on Rs89B1 and Rs89B1_ins activity. All reported values are relative to the specific activity performed under standard assay conditions. Error bar indicates standard deviation (n = 3). (a) The pH profiles were measured in ammonium acetate (-●-), phosphate (-■-), Tris-HCl (-▲-) buffers within their appropriate pH ranges. (b) Temperature profiles were measured at sodium phosphate buffer pH 7.0. The not detected conditions were indicated as n.d..

Glucosyltransferase activity was assessed using 2,3,4-THB as the acceptor substrate, with a reaction time of 5 min. The optimal pH for the glucosyltransferase fell within the range of 6.5 to 7.0 (Fig 3A). The preferred reaction temperature ranged from 30°C to 35°C, with no detectable activity observed above 50°C (Fig 3B). The influence of divalent cations was examined, including MgCl_2_, MnCl_2_, CaCl_2_, ZnSO_4,_ and EDTA at concentrations of 0.25, 0.5, and 1.0 mM under standard reaction conditions as described earlier. Both Rs89B1 and Rs89B1_ins exhibited glucosyltransferase activity even without divalent cations when EDTA was present. For Mg^2+^ and Mn^2+^ in Rs89B1, activity increased in a concentration-dependent manner at concentrations up to 1.0 mM, reaching 114.8% and 115.8%, respectively (Table 1). Rs89B1_ins displayed a similar trend, with activity values of 107.6% and 106.6% for 1.0 mM Mg^2+^ and Mn^2+^, respectively. In contrast, Zn^2+^ strongly inhibited the activity of both Rs89B1 and Rs89B1_ins. It is worth noting that Ca^2+^ did not affect enzyme activity. Divalent cation requirements and effects were similar between Rs89B1 and Rs89B1_ins, and no effect of the mutation was detected.

**Table 1:** Effects of divalent cations.

	% of relative activity					
	Rs89B1			Rs89B1_ins		
Cations	0.25 mM	0.5 mM	1.0 mM	0.25 mM	0.5 mM	1.0 mM
Mg^2+^	104.8	110.6	114.8	108.1	112.3	115.8
Mn^2+^	101.8	103.8	107.6	100.4	106.5	106.6
Ca^2+^	99.4	99.0	100.8	100.0	98.3	99.0
Zn^2+^	77.2	71.2	55.1	78.3	73.6	58.2
EDTA	99.6	99.5	99.8	99.5	99.7	99.1

Each divalent cation or EDTA was added to the enzyme reaction mixture, and the specific activity was measured. All reported values are relative to the untreated sample.

### Enzyme kinetic parameters of Rs89B1 and Rs89B1_ins

The enzyme concentrations used to calculate the enzyme kinetic parameters were determined in the enzyme concentration rage of the primary reaction, where the reaction rate was proportional to the enzyme concentration, using 0.2 mM 2,3,4-THBA and 1.0 mM UDP-glucose as substrates and changing the enzyme concentration (S4 Fig). The kinetic parameters for hydroxybenzoates of both Rs89B1 and Rs89B1_ins are presented in Table 2. Notably, both enzymes exhibited the lowest *k*_cat_/*K*_m_ value for 2,5-DHBA and the lowest *K*_m_ value for 3,4-DHBA. *K*_m_ and *k*_cat_/*K*_m_ values for 4-HBA were not significantly different between Rs89B1 and Rs89B1_ins. However, for 2,4- and 3,4-DHBA, *K*_m_ values of Rs89B1_ins were increased by 12% and 16%, respectively, from Rs89B1, while *k*_cat_/*K*_m_ values were decreased by 22% and 37%, respectively. A similar trend was observed for 2,4,6-THBA, where the *K*_m_ value of Rs89B1_ins increased 3.5-fold. The mutation comparatively reduced the enzyme’s substrate affinity, but it retained around 69% of the catalytic activity of Rs89B1. For 2,5-DHBA, Rs89B1_ins displayed a 62% higher *k*_cat_/*K*_m_ value and a 15% lower *K*_m_ value compared to Rs89B1, indicating enhanced catalytic activity due to the introduced mutation. Interestingly, under conditions of a large excess of UDP-glucose concentration, there was no significant change in *K*_m_ value for 2,3,4-THBA, but Rs89B1_ins exhibited a 25% higher *k*_cat_/*K*_m_ value than Rs89B1. Additionally, the enzyme kinetic parameters for the donor substrate, UDP-glucose, showed no significant differences between Rs89B1 and Rs89B1_ins. The mutation in the loop region altered the enzyme’s kinetic parameters, with varying effects on substrate affinity and enzyme kinetic activity depending on the substrate.

**Table 2:** Kinetic parameters of Rs89B1 and Rs89B1_ins.

	Rs89B1			Rs89B1_ins		
Substrate	*K*_m_ (μM)	*k*_cat_ (s^-1^)	*k*_cat_/*K*_m_ (mM⋅s^−1^)	*K*_m_ (μM)	*k*_cat_ (s^-1^)	*k*_cat_/*K*_m_ (mM⋅s^−1^)
4-HBA	232.6 ± 12.2	0.953 ± 0.013	4.1 ± 1.1	251.3 ± 15.4	0.696 ± 0.009	2.8 ± 0.6
2,4-DHBA	182.4 ± 4.2	2.061 ± 0.006	11.3 ± 1.4	212.4 ± 5.8	0.588 ± 0.003	7.2 ± 0.9
3,4-DHBA	70.4 ± 1.3	1.360 ± 0.001	19.3 ± 0.7	79.1 ± 2.3	1.179 ± 0.004	14.9 ± 1.9
2,5-DHBA	773.8 ± 1.6	0.572 ± 0.000	0.74 ± 0.1	663.2 ± 2.3	0.802 ± 0.001	1.2 ± 0.2
2,3,4-THBA	137.7 ± 2.0	3.098 ± 0.008	22.5 ± 0.4	128.2 ± 4.5	3.615 ± 0.018	28.2 ± 0.4
2,4,6-THBA	198.1 ±7.2	4.593 ± 0.008	23.2 ± 1.1	688.3 ± 14.6	10.984 ± 0.010	16.0 ± 0.7
UDP-glucose	53.8 ± 1.1	1.98 ± 0.010	36.8 ± 0.8	56.2 ± 0.6	1.93 ± 0.008	34.4 ± 1.4

## Discussion

A group of plant glucosyltransferases, predominantly operating in the cytoplasm, exhibit regioselective glucosylation, a feat challenging to replicate through chemical synthesis. Our study delved into the enzymatic properties of Rs89B1, a homolog of UGT89B1, and its mutants. UGT89B1 has been shown to glucosylate mainly to the hydroxyl group at the *para*-position of hydroxybenzoic and dihydroxybenzoic acids and to have some glucoylation activity at the *meta*-position of 2,5-dihydroxybenzoic acid [7]. Our findings indicate that Rs89B1 shares this glycosyltransferase activity, transferring glucose to hydroxybenzoates akin to UGT89B1. Notably, Rs89B1 also demonstrates glycosyltransferase activity toward THBAs. Benzoic acids without a *para*-hydroxyl group showed no activity, except for 2,5-DHBA. This suggests Rs89B1’s preference for adding glucose to the *para-*position (Fig 2). In addition, benzoic acids with *para*-hydroxyl groups tended to enhance enzymatic activity by the addition of *ortho*- or *meta*-hydroxyl groups.

Comparative analysis of enzyme kinetic parameters for various hydroxybenzoates showed that Rs89B1 exhibits the highest affinity for 3,4-DHBA. The presence of a *meta*-hydroxyl group in conjunction with the *para*-position significantly enhances substrate affinity (Table 1). In cases where the *para*-position lacks a hydroxyl group, such as 2,5-DHBA, the functional group at the *ipso*-position is believed to create steric hindrance, leading to decreased affinity [24]. Remarkably, Rs89B1_ins, a mutant with three amino acid residues inserted into the loop region away from the recognition site, displayed a similar substrate affinity pattern to Rs89B1. However, the affinity of Rs89B1 for 2,4,6-THBA was significantly reduced, possibly due to alterations in the flexibility of the mutagenized loop region [25]. In Rs89B1_ins, the affinity for 2,5-DHBA increased by approximately 15%, suggesting that the mutational site in Rs89B1 might alleviate aglycon steric hindrance, despite not directly impacting the substrate recognition site. Both Rs89B1 and Rs89B1_ins generally displayed increased catalytic activity as the number of hydroxyl groups on the benzene ring increased. When amino acid residues were inserted into the loop region, enzyme catalytic activity tended to vary depending on the substrate. An approximately 1.4-fold increase in activity was observed with 2,3,4-THBA, while the affinity remained comparable. In particular, 2,5-DHBA showed increased enzyme activity after the introduction of the insertional mutation in the loop region of Rs89B1, presumably due to the greater steric hindrance of the enzyme pocket. These results suggest that the mutations modestly affected the enzyme’s catalytic activity site. More detailed information on the interaction between enzyme active residues and substrates could be obtained by crystallographic analysis of the enzyme active pocket and structure correlation analysis.

This study underscores the potential for modifying enzyme catalytic activity by introducing mutations in a loop region distant from the recognition site. Importantly, the optimal reaction conditions remained consistent irrespective of the mutations, likely because the mutations were introduced in a manner that preserved the enzyme’s overall structure (Fig 3 and Table 1). It is worth noting that the mutated region in Rs89B1 is anticipated to be a non-conserved area, as indicated by multiple alignments of GT family 1 [26]. In most plant-derived GT family 1 enzymes, the pertinent loop region typically comprises approximately 30 amino acid residues. In contrast, bacterial GT family 1 enzymes consist of only a few amino acid residues. Notably, alterations in enzyme kinetic parameters were observed in Rs89B1 and Rs89B1_ins, and these changes did not align consistently with the position or number of hydroxyl groups on the hydroxybenzoates (Table 2). These findings further support the idea that the loop region investigated in this study plays a key role in the broad substrate specificity of plant-derived UGTs. Additionally, the introduction of enzymes with enzymatic activity fine-tuned by modifying the loop region of UGT into plants may enable the accumulation of specific secondary metabolites with glycosylated hydroxyl groups.

In conclusion, this study has unveiled the enzymatic properties of Rs89B1 and its glycosyltransferase activity toward THBAs. Moreover, our analysis of the mutant Rs89B1 revealed that, in addition to the position and number of hydroxyl groups on the benzene ring, mutations in the enzyme’s loop region can significantly impact catalytic activity. While traditional enzyme modification typically involves amino acid residues in the active site, recent attention has shifted toward introducing mutations in the enzyme’s exoskeleton [2, 27]. The results of this study highlight that introducing mutations in the loop region, even remotely from the enzyme’s recognition site, can comparatively change the affinity between the enzyme and its substrate. This represents a novel avenue for enzyme activity modification and holds promise as a target for future research in this field.

## Supporting information

S1 Fig Result of multiple sequence alignment for UGT89B1, homologous sequences from BLAST results, and Rs89B1_ins. Multiple sequence alignment was carried out by Clustal Omega. At the bottom of the sequence, * denotes a conserved sequence (identical),: denotes a conservative mutation, denotes a semiconservative mutation, and–denotes a gap. Underline indicates predicted PSPG box region. The region with low homology is indicated by the red shading, while the amino acid residues presumed to be involved in the recognition of UDP-sugars are indicated by the blue shading.
(PDF)

S2 Fig Predicted protein structures of Rs89B1 and Rs89B1_ins. The structures of Rs89B1 and Rs89B1_ins were predicted using AlphaFold2 with UGT89B1 (AlphaFoldDB, Q9C9B0) as the template structure. These structures were visualized using PyMOL. (a) Predicted structures. The red region indicates the gap residues between Rs89B1 and UGT89B1/Rs89B1_ins, while the blue region indicates the predicted binding or active amino acid residues. (b) An enlarged view of the mutation site. Gray indicates UGT89B1, magenta indicates Rs89B1_ins, and cyan indicates Rs89B1. The region colored red denotes the mutation sites.
(PDF)

S3 Fig SDS-PAGE (CBB staining) of purified recombinant Rs89B1and Rs89B1_ins. Lane M represents the marker (ExcellBand All Blue Regular Range Protein Marker, SMBIO Technology INC., Taiwan); lane 1 shows purified Rs89B1 (52.4 kDa), and lane 2 displays purified Rs89B1_ins (52.8 kDa). An arrow indicates the predicted molecular weights of the target proteins.
(PDF)

S4 Fig Enzyme initial velocities of purified recombinant proteins. (a) Time courses of Rs89B1 and Rs89B1_ins with 5.0 mM 4-HBA at 30°C and pH 7.0, using 0.1 mg/mL of proteins. (b) The effect of enzyme concentration on the initial velocities of Rs89B1 and Rs89B1_ins with 0.2 mM 2,3,4-THBA.
(PDF)

S5 Fig Enzymatic activities to compounds without hydroxyl groups in the *para-*position. Enzymatic activities for 2-HBA (a), 3-HBA (b), 2,3-DHBA (c), 2,6-DHBA (d), and 3,5-DHBA (e) were analyzed using HPLC.
(PDF)

S1 Table Detected *m/z* of LC elution peaks by mass spectrometry. (PDF)
