# Supplementary material for: Enzymatic properties of UDP-glycosyltransferase 89B1 from radish and modulation of enzyme catalytic activity via loop region mutation
Source: PLoS One. 2024 Feb 28;19(2):e0299755. doi: 10.1371/journal.pone.0299755 (PMC10901349; doi:10.1371/journal.pone.0299755)
Supplement: S1 Fig — Multiple sequence alignment was carried out by Clustal Omega. At the bottom of the sequence, * denotes a conserved sequence (identical),: denotes a conservative mutation, denotes a semiconservative mutation, and–denotes a gap. Underline indicates predicted PSPG box region. The region with low homology is indicated by the red shading, while the amino acid residues presumed to be involved in the recognition of UDP-sugars are indicated by the blue shading. (PDF) [file pone.0299755.s001.pdf]

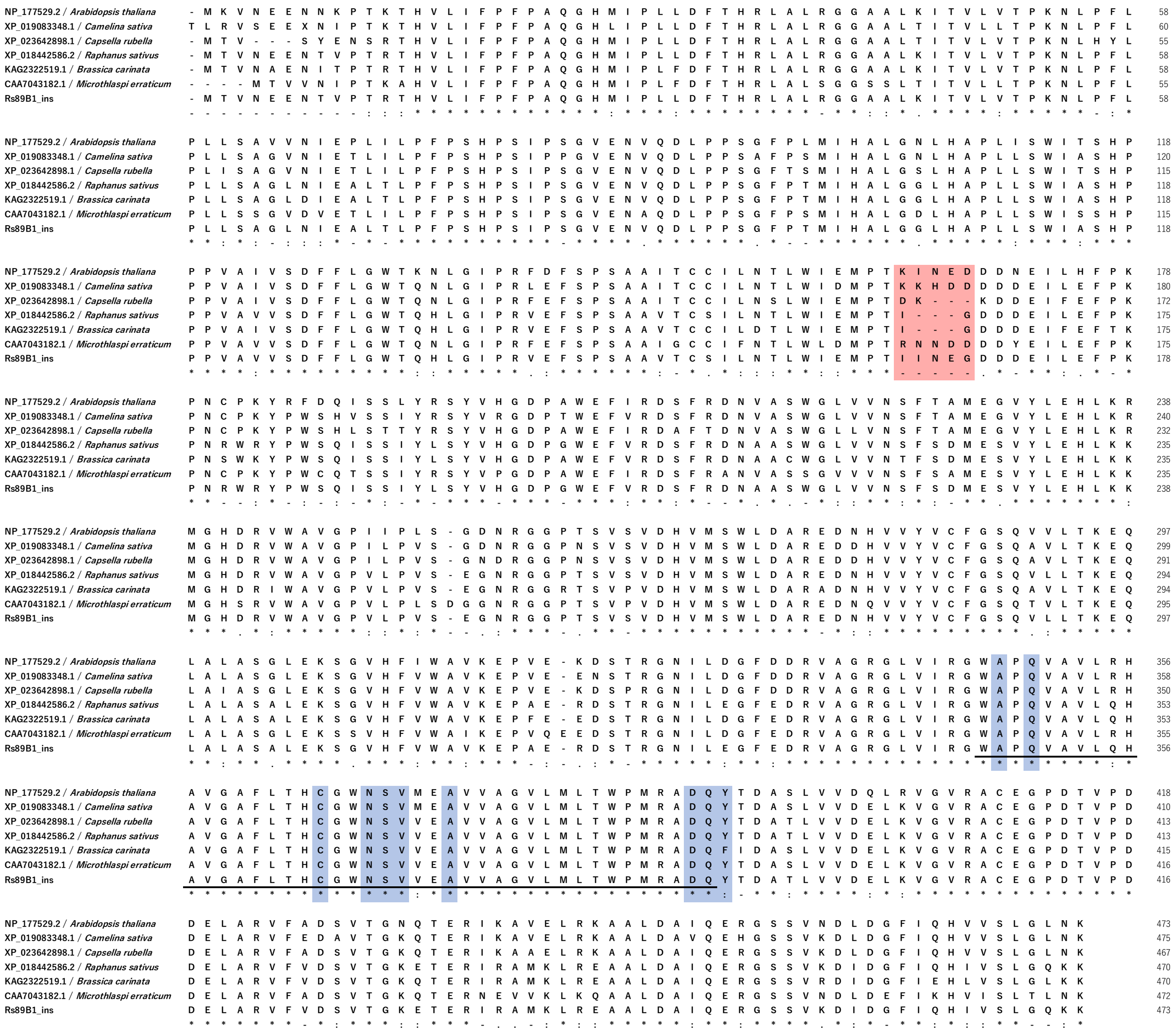

S1 Fig. Result of multiple sequence alignment for UGT89B1, homologous sequences from BLAST results, and Rs89B1_ins.

Multiple sequence alignment was carried out by Clustal Omega. At the bottom of the sequence, * denotes a conserved sequence (identical), : denotes a conservative mutation, ・ denotes a semiconservative mutation, and – denotes a gap. Underline indicates predicted PSPG box region. The region with low homology is indicated by the red shading, while the amino acid residues presumed to be involved in the recognition of UDP-sugars are indicated by the blue shading.
