# Supplementary material for: Enzymatic properties of UDP-glycosyltransferase 89B1 from radish and modulation of enzyme catalytic activity via loop region mutation
Source: PLoS One. 2024 Feb 28;19(2):e0299755. doi: 10.1371/journal.pone.0299755 (PMC10901349; doi:10.1371/journal.pone.0299755)
Supplement: S2 Fig — The structures of Rs89B1 and Rs89B1_ins were predicted using AlphaFold2 with UGT89B1 (AlphaFoldDB, Q9C9B0) as the template structure. These structures were visualized using PyMOL. (a) Predicted structures. The red region indicates the gap residues between Rs89B1 and UGT89B1/Rs89B1_ins, while the blue region indicates the predicted binding or active amino acid residues. (b) An enlarged view of the mutation site. Gray indicates UGT89B1, magenta indicates Rs89B1_ins, and cyan indicates Rs89B1. The region colored red denotes the mutation sites. (PDF) [file pone.0299755.s002.pdf]

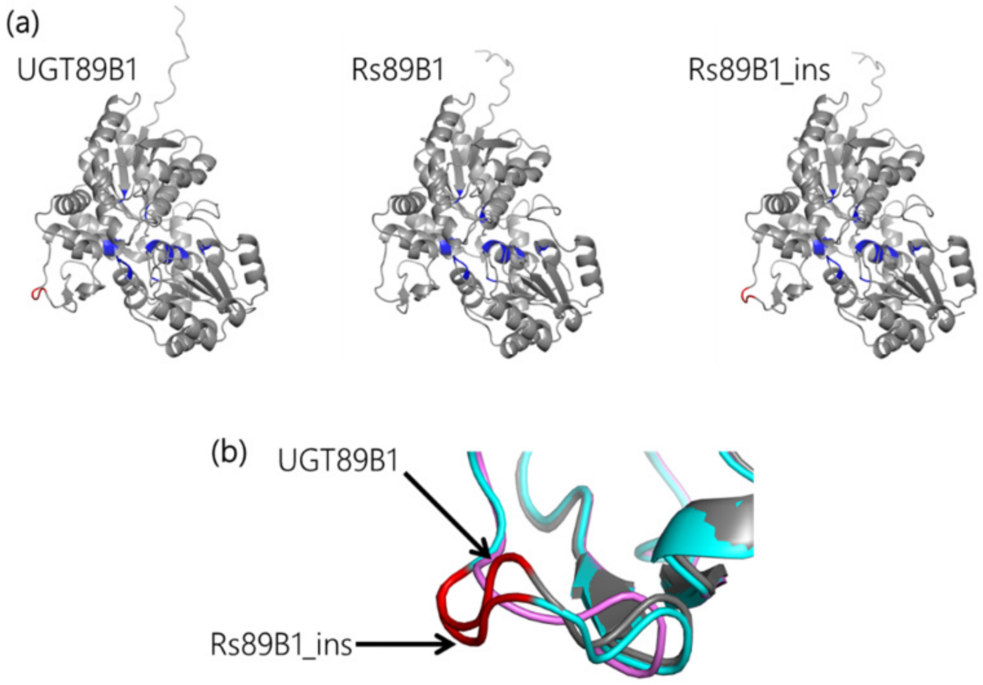

S2 Fig. Predicted protein structures of Rs89B1 and Rs89B1_ins.

The structures of Rs89B1 and Rs89B1_ins were predicted using AlphFold2 with UGT89B1 (AlphaFoldDB, Q9C9B0) as the template structure. These structures were visualized using PyMOL. (a) Predicted structures. The red region indicates the gap residues between Rs89B1 and UGT89B1/Rs89B1_ins, while the blue region indicates the predicted binding or active amino acid residues. (b) An enlarged view of the mutation site. Gray indicates UGT89B1, magenta indicates Rs89B1_ins, and cyan indicates Rs89B1. The region colored red denotes the mutation sites.
