# Supplementary material for: Enzymatic properties of UDP-glycosyltransferase 89B1 from radish and modulation of enzyme catalytic activity via loop region mutation
Source: PLoS One. 2024 Feb 28;19(2):e0299755. doi: 10.1371/journal.pone.0299755 (PMC10901349; doi:10.1371/journal.pone.0299755)
Supplement: S3 Fig — Lane M represents the marker (ExcellBand All Blue Regular Range Protein Marker, SMBIO Technology INC., Taiwan); lane 1 shows purified Rs89B1 (52.4 kDa), and lane 2 displays purified Rs89B1_ins (52.8 kDa). An arrow indicates the predicted molecular weights of the target proteins. (PDF) [file pone.0299755.s003.pdf]

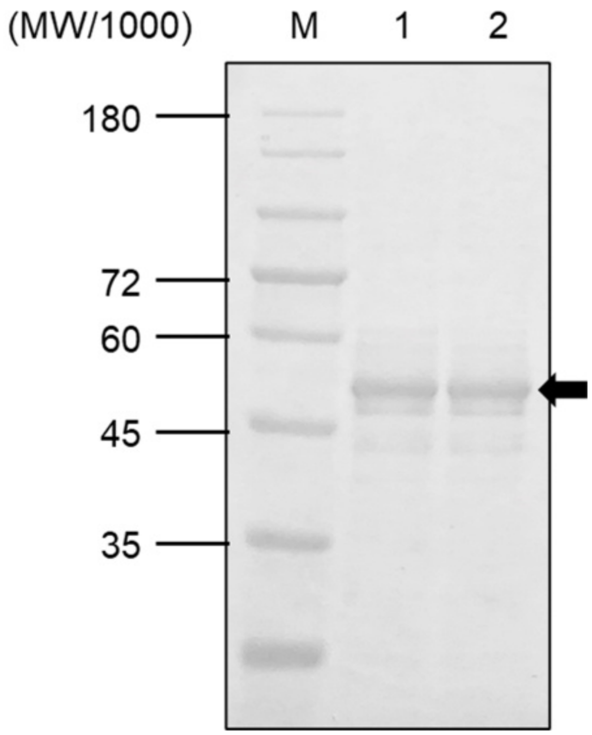

S3 Fig. SDS-PAGE (CBB staining) of purified recombinant Rs89B1and Rs89B1_ins. Lane M represents the marker (ExcellBand All Blue Regular Range Protein Marker, SMBIO Technology INC., Taiwan); lane 1 shows purified Rs89B1 (52.4 kDa), and lane 2 displays purified Rs89B1_ins (52.8 kDa). An arrow indicates the predicted molecular weights of the target proteins.
