# Supplementary material for: Enzymatic properties of UDP-glycosyltransferase 89B1 from radish and modulation of enzyme catalytic activity via loop region mutation
Source: PLoS One. 2024 Feb 28;19(2):e0299755. doi: 10.1371/journal.pone.0299755 (PMC10901349; doi:10.1371/journal.pone.0299755)
Supplement: S4 Fig — (a) Time courses of Rs89B1 and Rs89B1_ins with 5.0 mM 4-HBA at 30°C and pH 7.0, using 0.1 mg/mL of proteins. (b) The effect of enzyme concentration on the initial velocities of Rs89B1 and Rs89B1_ins with 0.2 mM 2,3,4-THBA. (PDF) [file pone.0299755.s004.pdf]

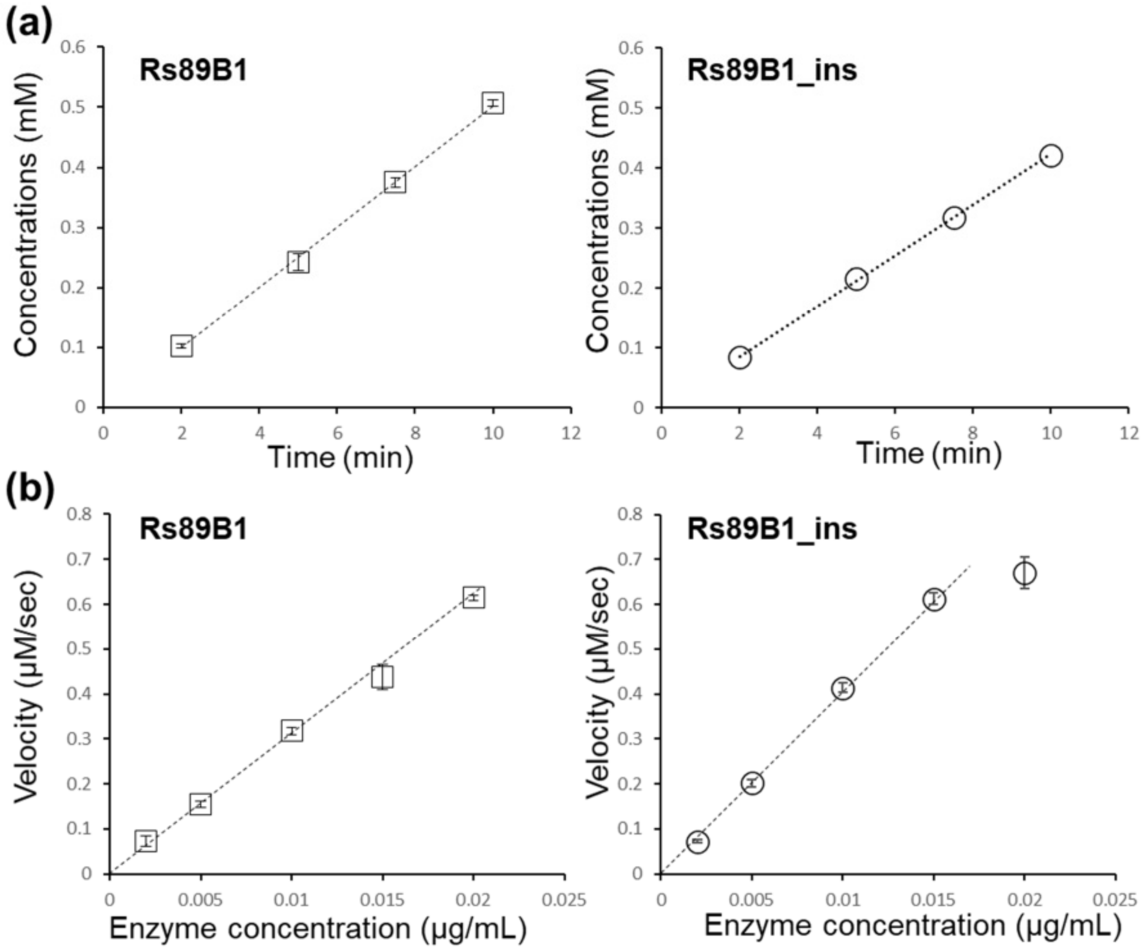

S4 Fig. Enzyme initial velocities of purified recombinant proteins. (a) Time courses of Rs89B1 and Rs89B1_ins with 5.0 mM 4-HBA at 30°C and pH 7.0, using 0.1 mg/mL of proteins. (b) The effect of enzyme concentration on the initial velocities of Rs89B1 and Rs89B1_ins with 0.2 mM 2,3,4-THBA.
