# Supplementary material for: Enzymatic properties of UDP-glycosyltransferase 89B1 from radish and modulation of enzyme catalytic activity via loop region mutation
Source: PLoS One. 2024 Feb 28;19(2):e0299755. doi: 10.1371/journal.pone.0299755 (PMC10901349; doi:10.1371/journal.pone.0299755)
Supplement: S5 Fig — Enzymatic activities for 2-HBA (a), 3-HBA (b), 2,3-DHBA (c), 2,6-DHBA (d), and 3,5-DHBA (e) were analyzed using HPLC. (PDF) [file pone.0299755.s005.pdf]

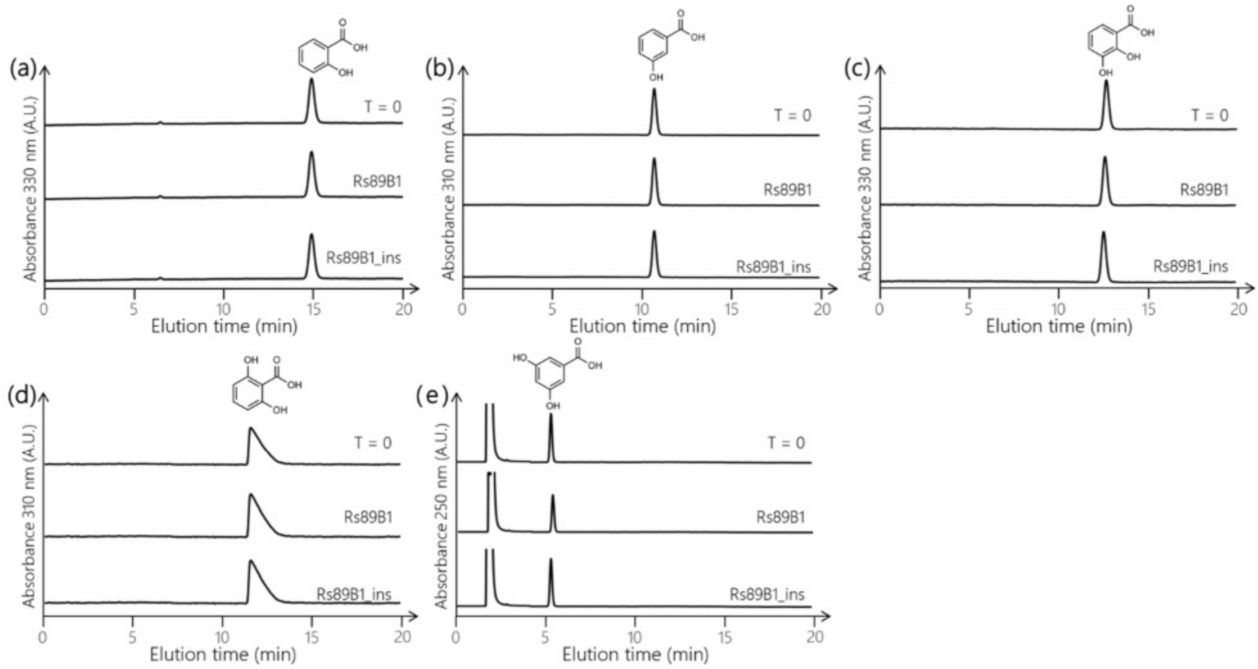

S5 Fig. Enzymatic activities to compounds without hydroxyl groups in the *para*-position.

Enzymatic activities for 2-HBA (a), 3-HBA (b), 2,3-DHBA (c), 2,6-DHBA (d), and 3,5-DHBA (e) were analyzed using HPLC.
