# Supplementary material for: Enzymatic properties of UDP-glycosyltransferase 89B1 from radish and modulation of enzyme catalytic activity via loop region mutation
Source: PLoS One. 2024 Feb 28;19(2):e0299755. doi: 10.1371/journal.pone.0299755 (PMC10901349; doi:10.1371/journal.pone.0299755)
Supplement: S1 Table — (PDF) [file pone.0299755.s006.pdf]

S1 Table. Detected *m/z* of LC elution peaks by mass spectrometry

	Substrate peak	Adduct	Product peak	Adduct	Delta *m/z*
4-HBA	*m/z* 139.0394	$[M + H]^+$	*m/z* 301.0923	$[M + H]^+$	*m/z* 162.0529
2,4-DHBA	*m/z* 155.0344	$[M + H]^+$	*m/z* 317.0872	$[M + H]^+$	*m/z* 162.0528
3,4-DHBA	*m/z* 155.0345	$[M + H]^+$	*m/z* 317.0873	$[M + H]^+$	*m/z* 162.0528
2,5-DHBA	*m/z* 155.0344	$[M + H]^+$	*m/z* 317.0873	$[M + H]^+$	*m/z* 162.0529
2,3,4-THBA	*m/z* 171.0294	$[M + H]^+$	*m/z* 333.0822	$[M + H]^+$	*m/z* 162.0528
2,4,6-THBA	*m/z* 171.0294	$[M + H]^+$	*m/z* 333.0824	$[M + H]^+$	*m/z* 162.0530

LC-MS analysis was performed using an Agilent 1260 LC with a 6530C Q-ToF MS system. Samples reacted with Rs89B1 were diluted 50 times, and 5 µL was analyzed. The separation conditions were identical to those used for HPLC analysis in the materials and methods section. The mass spectrometer was operated using a dual Agilent Jet Stream electrospray ionization (dAJS) source in positive ion mode. The ionization parameters included a nozzle voltage of 3,500 V, a nebulizer gas pressure of 55 psi, a drying gas temperature of 300ºC, a drying gas flow rate of 12 L/min, a sheath gas temperature of 200ºC, and a sheath gas flow rate of 11 L/min. Substrate and product peaks are indicated in Fig 2. The delta *m/z* was calculated by subtracting the *m/z* of the substrate peak from the product peak.
